# Has the frequency of violent behaviour increased during COVID-19 pandemic?

**DOI:** 10.1192/j.eurpsy.2021.845

**Published:** 2021-08-13

**Authors:** C.-A. Crisan, D. Ureche, C. Siserman

**Affiliations:** 1 Psychiatric Clinic 1, Emergency County Hospital Cluj-Napoca, Cluj-Napoca, Romania; 2 Neurosciences, Iuliu Hatieganu University of Medicine and Pharmacy Cluj-Napoca, Cluj-Napoca, Romania; 3 Community Medicine, Iuliu Hatieganu University of Medicine and Pharmacy Cluj-Napoca, Cluj-Napoca, Romania; 4 Community Medicine, Legal Institut Cluj-Napoca, Cluj-Napoca, Romania

**Keywords:** interpersonal violence, crime, Covid-19 pandemic, psychiatric disturbances

## Abstract

**Introduction:**

Covid-19 pandemic represents o very stressfull period for many individuals. Intimate partner violence is typically experienced by women but can also be experienced by men. During quarantine due to the Covid-19 pandemic, the risk for domestic violence increased.

**Objectives:**

The aim of this study was to compare domestic violence durind two different periods - 14.03.2019-30.09.2019 and the same period of the year 2020.

**Methods:**

We analyzed the data base of the Legal Institut from Cluj-napoca and we selected the cases with domestic violence.

**Results:**

The violent behaviour incresed durind 2020. Due to the feelings of frustration and agitation, agression arises with possible trasgenerational transmission of trauma and violence.

**Conclusions:**

Taking into consideration that Covid19 pandemic is a very stressfull period for all individuals, a need of programms aimed to prevent acts of domestic violence and to achieve accurate assessment of multiple domains of abuse (psychological, physical, sexual) provided by psychologists, psychiatrists, social and legal services emerged.

